# Intrauterine device (IUD) migration in cesarean delivery scar: What to do with the niche?

**Published:** 2020-01-24

**Authors:** A Verest, E Borwski, I Cadron, S Van Calenbergh, R Vanspauwen

**Affiliations:** Dept. of Obstetrics and Gynecology, Algemeen Ziekenhuis Turnhout, Steenweg op Merksplas 44, 2300 Turnhout, Belgium;; Faculty of medicine, KU Leuven Gasthuisberg, Herestraat 49, 3000 Leuven, Belgium.

**Keywords:** Cesarean scar defect, niche repair, IUD, laparoscopy, hysteroscopy, residual myometrium

## Abstract

**Background:**

The presence of a niche after cesarean section is a common and mostly asymptomatic finding. However, it can cause symptoms or result in impaired fertility or obstetric complications in following pregnancies. At present there is no uniform consensus on when to treat and which way of repair is most suitable. The aim of this systematic review of literature was to provide an overview of current knowledge about cesarean scar niches and about the modalities of niche repair.

**Methods:**

On the second of January 2019 Pubmed and Cochrane databases were searched for relevant studies published until December 2018. Search terms were cesarean scar defect, niche, niche repair. As combination key words `hysteroscopy ´, `laparoscopy ´ and `vaginal repair ´ were used.

**Results:**

Eight articles were included in this review. The publications were very heterogeneous. Most of them stated that hysteroscopic niche repair with resection of the lower (and upper) rim is suggested for abnormal uterine bleeding. In symptomatic women who wish to conceive, different authors suggest laparoscopic niche repair with double layer closure to increase myometrial thickness. Also, one report on vaginal repair was included, none of the included patients had child wish. Nothing was reported on residual myometrial thickness after surgery.

**Conclusion:**

The current literature is not sufficient to draw strong conclusions on what to do about cesarean scar niches, yet, they justify the role of hysteroscopic as well as laparoscopic niche repair dependent on different pre- operative factors. We conclude that further large randomized controlled trials are necessary.

## Background

### Case report

A 38-year-old female presented to the emergency department with severe low abdominal pain, irradiating to the right iliac fossa. The continuous pain started two days before, after sexual intercourse and was progressively intensive. The patient previously underwent three cesarean sections, the first two were performed in Kenia because of cephalopelvic disproportion and fetal distress. An elective repeat cesarean section was performed in our clinic at 39 weeks of gestation in her third pregnancy in 2012. Two years later a levonorgestrel intrauterine device was inserted for contraception and was correctly positioned at level of the uterine fundus documented by transvaginal ultrasound three months after placement.

The patient was amenorrhoeic since two months after insertion of the intrauterine device (IUD).

The IUD had been in situ for three years without any symptoms of pain.

When clinically examined, the patient was hemodynamically stable and afebrile. There was diffuse abdominal tenderness on palpation, especially in the right iliac fossa, with no associated clinical signs of peritonitis. On speculum examination, a normal cervix was visualized but the threads of the IUD could not be seen at level of the external cervical ostium.

**Figure 1:**
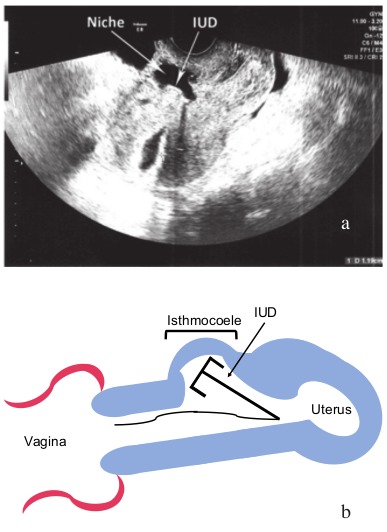
— Figure 1a-1b: Ultrasound of the uterus: Uterus in anteversionflexion. Isthmocoele located in the anterior wall, free fluid in the cavity. IUD positioned in the isthmocoele. b: schematic drawing of uterus with IUD migrated into isthmocoele.

Transvaginal ultrasound revealed a uterus in anteversion flexion with an IUD migrated into a cesarean scar niche (18x17x26 mm), or isthmocoele (another name for large niche) with the lower part of the IUD still remaining at the level of the uterine cavity, which was distended by fluid (12 mm) (Figure 1).

The ovaries were normal and a limited amount of free fluid in the pelvis (11x20 mm) was seen.

The blood tests were unremarkable and demonstrated a negative HCG, Hemoglobin (Hb) 12.7 g/dL, White Blood Cell (WBC) count of 3.9x10 6 /L, C-reactive protein (CRP) 1.6 mg/L.

Because of these clinical findings a hysteroscopy was performed. Upfront we planned to reposition the IUD, but during surgery the largeness of the isthmocoele seemed favorable for remigration of the IUD so there was decided to remove the IUD. Because the patient was already suffering from pain, this was performed under general anesthesia instead of the conventional in-office hysteroscopy procedure. The hysteroscopy confirmed the sonographic findings of the IUD located in the isthmocoele (Figure 1). The IUD was easily removed hysteroscopically by grasping the strings which were still at level of the uterine cavity.

**Figure 2:**
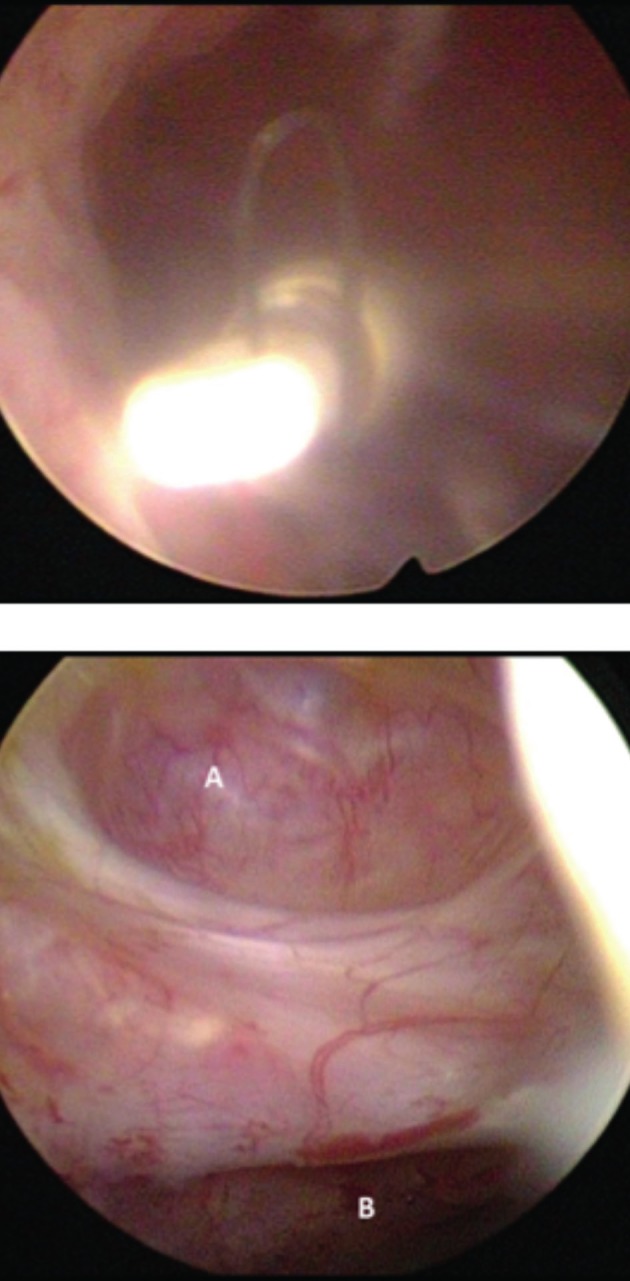
— Above: IUD located in the isthmocoele. Below: Border between isthmocoele (A) and uterine cavity (B).

Further inspection showed that the isthmocoele was located at the level of the previous cesarean delivery scar which was dehiscent and was still covered by uterine serosa without complete perforation (Figure 2).

Above the dehiscence a normal uterine cavity was visualized with normal endometrium and two normal tubal ostia. Concomitantly, a cystoscopy was performed to exclude vesical perforation.

This revealed some bulging of the isthmocoele on the posterior wall of the bladder but no perforation.

After removal of the IUD, her pain was immediately relieved and the patient was discharged the same day. An oral contraceptive was prescribed and, at postoperative control, six weeks later she was still asymptomatic.

Because the lack of symptoms and fulfilled child wish a conservative management was maintained and no niche repair was performed. Although we didn ´t perform a repair in this patient, we did laparoscopic and hysteroscopic repair in our centre before, this case inspired us to review the current knowledge about niches and their repair. Our goal was to have an evidenced-based approach for repair of niches in similar cases.

## Introduction

The purpose of this literary review was to make an overview of current knowledge about cesarean scar niches and the different possible modalities of niche repair. Over the last years, an increasingly number of cesarean sections has been performed and the prediction of experts is that this number will further increase. A cesarean scar defect is not uncommon after one or more cesarean sections and therefore every gynecologist will be confronted with this pathology. Migration of an IUD has been reported in literature and even migration through a niche into the bladder has occurred (Gyasi-Sarpong, 2016).

Diagnosis of uterine niche is mostly made with ultrasound. A cesarean scar defect or niche is defined as an anechoic space at the presumed site of the cesarean section scar where the myometrium is thinner than in other parts of the uterus. If the niche is large, sometimes the name isthmocoele is used (Bij de Vaate et al., 2011). Although a niche is mostly asymptomatic it sometimes can cause symptoms such as menstrual bleeding disorders like postmenstrual spotting, chronic pelvic pain and secondary infertility. In case of pregnancy it can lead up to obstetrical complications such as scar dehiscence, scar pregnancy and an adherent placenta as seen in placenta increta or accreta (Morris, 1995; Monteagudo et al., 2001; Bij de Vaate et al., 2014).

Van der Voet et al. (2014) performed a prospective cohort study in 2013 to investigate the prevalence of niches in women after cesarean section and their relationship with complaints of postmenstrual spotting and urinary incontinence. They included women who underwent one to three cesarean sections and evaluated them by transvaginal ultrasound and gel instillation sonohysterograpy (GIS) 6-12 weeks after most recent cesarean section. The women completed a questionnaire 6-12 weeks, 6 months and 12 months after cesarean section. In women with one cesarean section they found a niche in 62%, compared with 68.2% of women with two cesarean sections and 77.8% of women with three cesarean sections. Women with residual myometrium at the site of the uterine scar measuring <50% of the adjacent myometrial thickness had postmenstrual spotting more often than women with a residual myometrial thickness of >50% of the adjacent myometrial thickness. Urinary incontinence was not related to the presence of a niche in this study.

Besides postmenstrual bleeding, cesarean niches are also associated with chronic pelvic pain and secondary infertility. A histopathological study of hysterectomy specimens with Cesarean section scars of Morris et al. (1995) proposed a possible mechanism for the chronic pelvic pain and dyspareunia. They stated that lymphocytic infiltration, iatrogenic adenomyosis confined to the scar and the distortion of the lower uterine segment could contribute to these symptoms.

Currently, the mechanism leading to subfertility is not exactly known. Florio et al. (2012) proposed a hypothesis about possible causes of secondary subfertility with no other reasons for subfertility than the presence of an isthmocoele or scar niche. Persistence of the menstrual blood after menstruation in the cervix may negatively influence the mucus and sperm quality, obstruct sperm transport through the cervical canal and interfere with embryo implantation. Different studies report better results of fertility treatment after repairing the scar niche (Fabres et al., 2005; Gubbini et al., 2011; Florio et al., 2012). However, the association between a cesarean scar defect and infertility had never been proved because of absence of large randomized controlled trials on this specific topic.

Besides, the already known risks of cesarean section such as uterine rupture, placenta previa, ectopic pregnancy, infertility and intra-abdominal adhesions there seem to be other obstetrical risks which are specifically associated with a cesarean scar defect. Timor-Tritsch and Monteagudo (2012) stated that scar pregnancy and pathologically adherent placenta are raising in frequency together with the raise in amount of cesarean pregnancies. Both entities are associated with significant life- threatening complications such as hemorrhage, shock, maternal and fetal death. Because of the growing amount of cesarean sections performed and the possible high cost and life-threatening complications the question for prevention and consensus about treatment modality is raising.

Possible factors that could play a role in niche development include a very low incision through cervical tissue, inadequate suturing technique or patient-related factors that impair wound healing or increase inflammation or adhesion formation (Vervoort et al., 2015).

## Methods

### Search strategy

The databases used for literature search were Pubmed and Cochrane Library both, until December 2018. The keywords used were ‘cesarean scar defect’ and ‘niche’. After searching these two databases, the key words led to 33 publications on Pubmed and 5 on Cochrane library.

**Figure 3:**
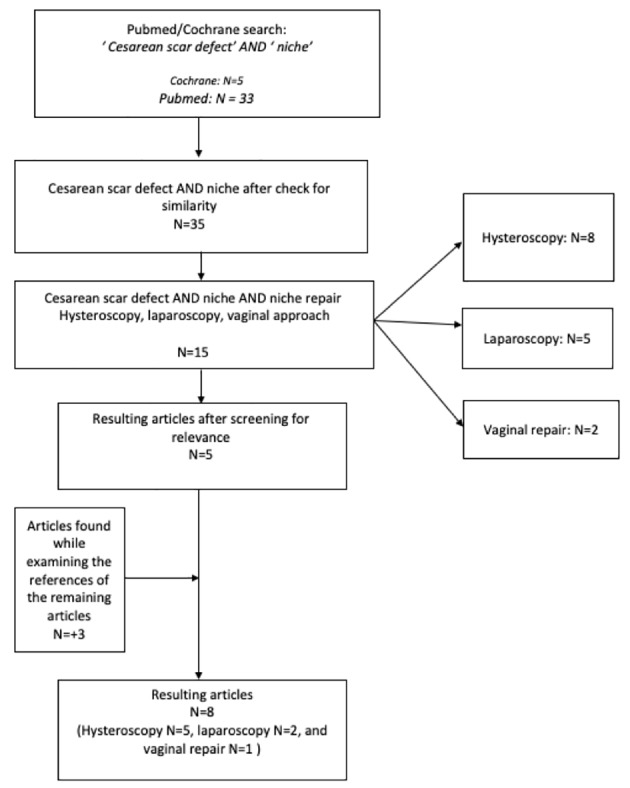
— Flow diagram of the literature search.

When these publications were checked for similarity, 35 different articles could be included. To narrow our findings, we added `niche repair ´ to the key words and ‘hysteroscopy’, ‘laparoscopy’ and ‘vaginal repair’ were used as combination key words. This search gave a total of 15 different publications in Pubmed (8 for hysteroscopy and 5 for laparoscopy and 2 for vaginal repair) and no hits in Cochrane library. The flow diagram of the literature search is presented in Figure 3.

### Eligibility criteria

These 15 articles were screened on availability of full text, the content and relevance of title and abstract by one reader. The articles that focused on the way of repair of the niche and outcomes (effect on abnormal uterine bleeding, pain relief, sexual function, quality of life (QOL), and surgical, anatomic, fertility, or pregnancy outcome) were included. The studies that described case reports, or combined niche investigation with other treatments (e.g. hysteroscopic sterilization) were excluded. The eligible 5 articles remained.

While examining the references of these 5 publications, 3 more articles were added.

In the end, 8 studies remained. The flow diagram of search strategy is shown in figure 3.

## Results

At present there is no uniform consensus on when to treat and on which modality of treatment is best: few authors suggest a transvaginal repair, but more common a minimally invasive approach by means of hysteroscopy or laparoscopy is recommended. Our review of literature found 8 articles of interest (Table I). Firstly, we discussed the studies about hysteroscopic repair.

Gubbini et al. (2011) performed a prospective study to evaluate the effect of hysteroscopic repair in restoring fertility. They included 41 patients with diagnosis of isthmocoele and secondary infertility. They all had symptoms of abnormal uterine bleeding or suprapubic pain and other possible causes of infertility were excluded. All patients were treated with hysteroscopic repair of the niche. Afterwards, they all became pregnant within 12-24 months after surgery. Of those women 37 delivered by cesarean section and 4 had a spontaneous miscarriage in the first trimester. All patients were relieved of their symptoms.

Fabres et al. (2005) performed a retrospective review of 52 premenopausal women with symptoms of postmenstrual bleeding and diagnosis of a niche by ultrasound or hysteroscopy. They included 24 patients. Postoperative follow up was 2 years in 21 patients and at least 14 months in the other three patients. Asymptomatic patients without abnormal uterine bleeding after surgery accounted for 84% of patients.

Raimondo et al. (2015) performed one of the first prospective studies in this matter. They included 120 symptomatic premenopausal women with isthmocoele diagnosed by ultrasound and office hysteroscopy. They performed a hysteroscopic repair in all patients. Abnormal uterine bleeding disappeared in 80% of patients, 7% of patients reported improvement of symptoms and 13% did not obtain any relief.

Gubbini et al. (2008) also set up a prospective study with 26 patients with one or more cesarean deliveries to assess the effectiveness of a hysteroscopic surgical technique to correct this anatomic defect and in that way eliminate the symptoms. All of them underwent resectoscopic correction of the isthmocoele. In all 26 patients symptoms were resolved. Seven out of nine (7/9) patients with secondary infertility became pregnant.

The Dutch team with Vervoort et al. (2018) performed a multicenter randomized trial comparing hysteroscopic niche resection with no intervention. As primary outcome they noted the number of days with postmenstrual spotting during one menstrual cycle 6 months after randomization.

They randomized 52 women in the intervention group and 51 women in the expectant management group. All women had ≥3 mm residual myometrium. Baseline median number of days of postmenstrual spotting was 8 days. Six months after randomization the intervention group reported a median of 4 days postmenstrual spotting whether the no intervention group reported 7 days (p=0.04). Also, discomfort was reported as 2 on a scale of 10 in the intervention group and 7 in the control group (p=0.02).

The laparoscopic repair approach was also investigated in different studies.

Donnez et al. (2017) reported on laparoscopic niche repair where the niche was removed (by laser or by coagulation) and the uterine scar was closed in two new layers. They found a significantly increase in the mean thickness of the myometrium. In this study, no obstetrical complications were seen. The patients who became pregnant after the repair all delivered by elective cesarean section at 38-39 weeks of pregnancy. 

The group of Vervoort et al. (2018) also performed a prospective trial with laparoscopic repair. They included 101 women with either dysmenorrhea, intrauterine fluid accumulation and/or difficulties with embryo transfer due to distorted anatomy with all a residual myometrium <3 mm. All women undergoing laparoscopic niche repair filled in questionnaires after 6 months and the niche was measured by ultrasound at baseline, 3 and 6 months after randomization. The primary outcome was reduction of the main problem 6 months after the intervention. One of the secondary outcomes was niche measurement. In 80 women (79.2%) the main problem was improved or resolved. The residual myometrium was increased significantly at follow up.

More authors state that, in women who wish to conceive, laparoscopic repair of the niche results in an increased thickness of the myometrium and is the preferable way to repair (Nezhat et al., 2016).

The only report on vaginal repair included was from Luo et al. (2012). They included 42 patients retrospectively. All patients reported abnormal uterine bleeding, postmenstrual spotting of prolonged bleeding during menstruation. Diagnosis was made by ultrasound and only if necessary with hysteroscopy. For 92.9% (39/42) of patients, relieve of symptoms was reported. After surgery, the median duration of menstruation was 6 days (range, 4–15 days), which was significantly shorter than before surgery (median, 13.3 days; range, 8–22 days; p<0.001). None of the patients reported child wish after surgery.

## Discussion

Several studies who investigated hysteroscopic repair, showed a good relief of symptoms with a higher chance on pregnancy. The laparoscopic surgical repair also had good results for symptom relief and increased myometrial thickness.

A systematic review of literature of Van der Voet et al. (2014) included twelve studies.

Two studies included in this Dutch review reported on vaginal repair. Although the methodological quality of the selected papers was considered to be moderate to poor, they reported relieve of symptoms in 86.9% of patients with a vaginal repair.

Only the report of Luo et al. (2012) was included in our review because of availability of full text. Luo included 42 patients retrospectively of whom 39 (92.9%) had relieve of symptoms. However, none of the patients had child wish after surgery and the median duration of menstruation postoperatively was still 8 days with a range between 4-15 days. Myometrial thickness was not reported.

The review of Van der Voet et al. (2014) included six studies that reported on hysteroscopic niche resection as a treatment for abnormal uterine bleeding. In the hysteroscopic niche resection the lower rim (and in some studies also the upper rim) of the niche is removed to modify the edges because of the hypothesis that abnormal uterine bleeding originates in collected blood in the niche during menstruation. It is suggested that this restores the subfertility but has no influence on the obstetric complications such as scar dehiscence because of the persistent thin myometrium.

A recent review of literature of Vervoort et al. (2015) and a study from Kataoka et al. (2016) both showed less niches in the cesarean section scar when double-layer interrupted sutures were used.

Full uterine thickness closure resulted in thicker mean myometrial thickness and less niches in follow up. This was not further explored in this review.

Florio et al. (2012) also reported on different possibilities of treatment and the effect on fertility. They performed a small literary review and state that hysteroscopic repair is the better choice for treatment yielding good therapeutic response with minimal invasiveness.

## Conclusions

Niches are a very common phenomenon after cesarean section. Although frequently asymptomatic they can cause symptoms and serious complications. It is very important that clinicians are aware of the potential existence of a niche and the possibilities to repair them.

We performed a systematic review of literature on this subject. There are a few larger, well powered and correctly performed studies who suggest that for hysteroscopic repair a minimal residual myometrial thickness of ≥3 mm is necessary. In women with thinner myometrial thickness a laparoscopic repair can be considered. Other have concluded that if >50% of the myometrium thickness is conserved hysteroscopic repair could be considered in patients with secondary subfertility without any other reasons for the failure to conceive. 

To our best knowledge it remains arbitrary which criteria for treatment should be used, some suggest a remaining myometrial thickness less than 2-3 mm is an indication, others compare the remaining thickness of the myometrium at level of the niche to the thickness of the adjacent myometrium. Also, clinical findings at the time of the cesarean section (e.g. fenestration or dehiscence) should be taken into account.

Overall, most of the studies presented a rather small sample size and variability for the defined outcome. Therefore, this review lacks firm conclusions and highlights the need for further research in large randomized controlled trials given the increasing prevalence of cesarean scar defects.
